# Risk Benefit Analysis of Routine Thymectomy for Differentiated Thyroid Cancers: A Systematic Review

**DOI:** 10.1055/s-0041-1736669

**Published:** 2021-12-15

**Authors:** Pallvi Kaul, Priyanka Kaul, Dharma Ram Poonia, Ashish Jakhetiya, Vipin Arora, Pankaj Kumar Garg

**Affiliations:** 1 Department of ENT and Head Neck Surgery, All India Institute of Medical Sciences, Rishikesh, Uttarakhand, India; 2 Department of General Surgery, Government Medical College, Jammu, India; 3 Department of Surgical Oncology, All India Institute of Medical Sciences, Jodhpur, India; 4 Department of Surgical Oncology, Geetanjali Medical College Hospital, Udaipur, Rajasthan, India; 5 Department of ENT and Head Neck Surgery, University College of Medical Sciences and Guru Teg Bahadur Hospital, University of Delhi, Delhi, India; 6 Department of Surgical Oncology, Shri Guru Ram Rai Institute of Medical and Health Sciences, Dehradun, India

**Keywords:** Head And Neck Cancer, Thyroid neoplasms, Central compartment node dissection, Thyroidectomy, Thymectomy

## Abstract

**Background** Central compartment lymph node dissection (CLND) is a part of the surgical management of differentiated thyroid cancer (DTC). Therapeutic CLND is done to address clinically significant central compartment nodes in patients with DTC, while prophylactic CLND is performed in the presence of high-risk features in the absence of clinically significant neck nodes. Removal of thymus—unilateral or bilateral—during CLND to achieve complete clearance of level VI and VII lymph node stations and address thymic metastasis is debatable.

**Objective** The present systematic review was conducted to summarize the evidence, delineating the role of thymectomy during CLND in patients with DTC.

**Methods** Electronic databases of PubMed, Embase, and Cochrane were searched from their inception to July 2020 using keywords—thyroid neoplasms or tumors, thyroidectomy, and thymectomy—to identify the articles describing the role of thymectomy during CLND in DTC. A pooled analysis of surgicopathological outcomes was performed using metaprop command in STATA software version 16.

**Result** A total of three studies and 347 patients—total thyroidectomy (TT) with bilateral thymectomy in 154, TT with unilateral thymectomy in 166, and TT alone in 27 patients with DTC—were included in the systematic review. The pooled frequency of thymic metastasis was a mere 2% in patients undergoing either unilateral or bilateral thymectomy. The routine addition of thymectomy does not result in better lymph node clearance. Unilateral and bilateral thymectomy were associated with high chances of transient hypocalcemia (12.0% and 56.1%, respectively).

**Conclusion** Routine thymectomy is not warranted during CLND, considering minimal oncological benefit and high risk of postoperative hypocalcemia.

Thyroid cancer is a leading endocrine malignancy with differentiated thyroid cancers accounting for 90% of cases. GLOBOCAN 2018 documented 567,233 new cases of thyroid cancer and 41,071 deaths annually. 1 Central compartment lymph node dissection (CLND) is an integral part of surgical management of differentiated thyroid cancers (DTC), depending upon the anticipated risk of metastasis based on various risk factors. 2 However, CLND is associated with significant postoperative morbidity due to the presence of many vital structures in a relatively narrow anatomical space.

There are several inconsistencies among the international guidelines regarding the inferior limit for CLND in thyroid cancer. The American Thyroid Association (ATA) management guidelines, published in 2015, specify CLND to target level VI station lymph nodes. 3 However, the ATA's consensus statement on terminology defines the boundaries for the CLND as follows: hyoid bone superiorly, the innominate artery on the right and corresponding axial plane on the left side inferiorly, medial aspect of the carotid sheath laterally, prevertebral fascia posteriorly, and the superficial layer of the deep cervical fascia anteriorly ( Fig. 1 ). 4 This equates CLND to incorporate both level VI and VII station lymph nodes. Furthermore, AJCC (American Joint Committee on Cancer) 7th edition recommended the involvement of level VII nodes to be staged as N1b, while AJCC 8th edition included level VII metastatic nodal disease as N1a category. However, both staging guidelines consider them regional, mandating level VII lymph node clearance.

**Fig. 1:**
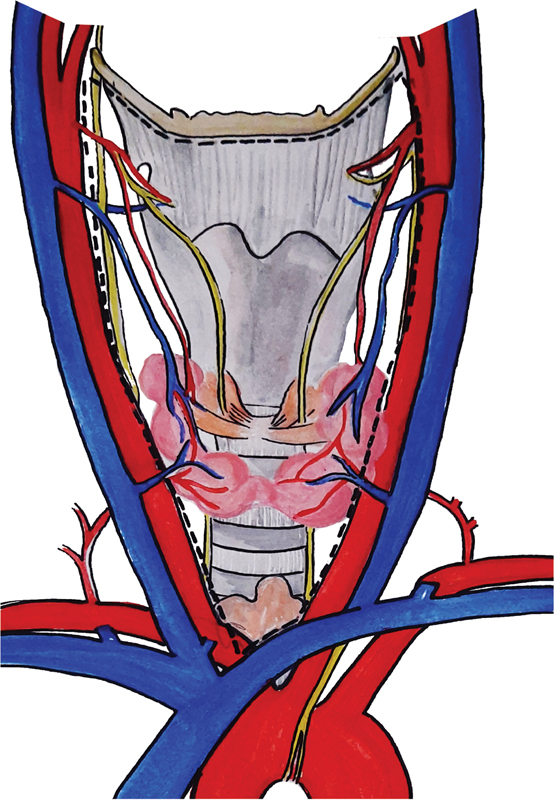
Surgical boundaries of the central compartment node dissection (dotted lines)

The thymus is anatomically located in the superior mediastinum and is encountered during level VII lymph node dissection. Should thymectomy be done routinely as a part of CLND to achieve good level VII clearance has not been addressed properly in the literature. The rationale of performing thymectomy in CLND is as follows: (a) it permits better lymph node clearance, as the thymus gland, especially its superior horns, lies within the anatomical confines of the central compartment, and (b) it entails extirpation of thymic metastasis if any. However, thymectomy performed as a part of CLND poses a significantly high risk of postoperative hypocalcemia, as the upper poles of the thymus as well as the inferior parathyroid glands share a common embryological origin from the endoderm of III pharyngeal pouch and lie close to each other in the paratracheal area within the domains of surgical boundaries. 5

The present systematic review aimed to analyze the risk-benefit of the routine thymectomy in CLND for DTC. A pooled analysis of previously conducted studies comparing morbidity associated with bilateral versus unilateral versus no thymectomy to determine the optimal extent of CLND was also performed.

## Methods

The systematic review of the literature was conducted following the Preferred Reporting Items for Systematic Review and Meta-Analysis Protocols (PRISMA-P) guidelines. The protocol of this systematic review was registered in the International Prospective Register of Systematic Reviews (PROSPERO) with the registration number CRD42020186741.

### Search Strategy

A thorough literature search was conducted using the electronic databases of MEDLINE (PubMed), Embase (Ovid), and Cochrane Library (Wiley) of the systematic review. A complete search strategy was developed following a consensus among the coauthors in collaboration with an external expert. The search strategy used variations in keywords—thyroid neoplasms or tumors, thyroidectomy, and thymectomy—found in the title, abstract, or keyword fields to retrieve articles referring to the role of thymectomy during CLND in DTC. Filters (humans and English) were applied to refine the search, and the articles published since the inception until July 2020 were included in the analysis. Single case reports/editorials/commentaries were not included in the review. The abstracts of the articles retrieved were screened for their relevance to our topic of study. The full text of the pertinent articles was obtained and evaluated. The references of these articles were also evaluated to look for any relevant studies. EndNote, version 8 (Clarivate Analytics) was used to facilitate the search process.

### Data Extraction

Two authors (P.K. and P.K.G.) searched the electronic databases and screened all the titles and abstracts from the selected articles. Any disagreement was resolved by the consensus among the authors. The full texts of the selected articles were analyzed by the three authors (P.K., P.K.G., and D.R.P.). The relevant information was extracted using a predefined data extraction sheet. The information collected included study location, year of publication, study design, sample size, clinicopathological details, and treatment outcomes of the patients included in the study.

### Statistical Analysis

All the relevant data was entered on the Microsoft Excel sheet and analyzed. A pooled analysis of surgicopathological outcomes was performed using metaprop command in the STATA software version 16.

## Results

**Fig. 2:**
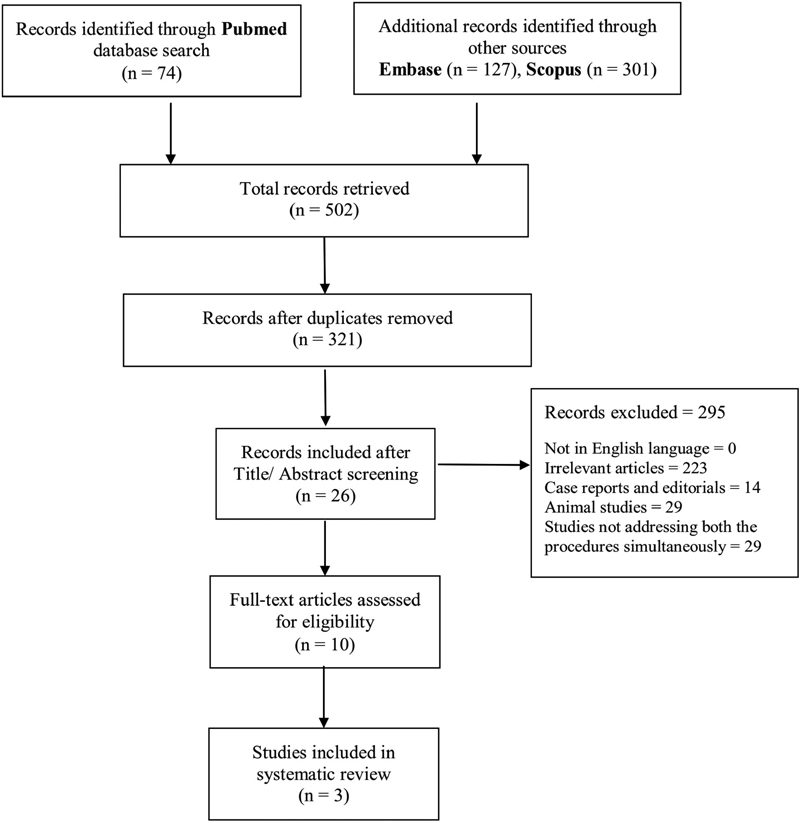
PRISMA chart

An initial database search of PubMed, Embase, and Cochrane using the stated keywords yielded 74, 127, and 301 articles, respectively. A total of 321 articles were identified after the removal of the duplicates. The search results were narrowed down to 26 after screening the titles. The abstracts of these articles were reviewed and a total of 10 full-text articles were assessed for eligibility after removing all the studies not addressing both the procedures simultaneously. After a thorough evaluation, we found three articles 6 7 8 that fulfilled the inclusion criteria, which were included in the systematic review ( Fig. 2 PRISMA chart). There was one randomized controlled trial and two retrospective studies addressing this issue.

The studies included were heterogeneous, concerning the inclusion and exclusion criteria and patient population. A formal assessment of the quality of the studies and publication bias could not be undertaken due to the presence of a few studies. Three studies satisfying the inclusion criteria were included in the present review and have been enlisted in Table 1 . A pooled analysis of the 347 patients included in these studies was carried out. Among the 347 patients, 154 underwent total thyroidectomy (TT) with bilateral thymectomy, while TT with unilateral thymectomy was performed in 166. Total thyroidectomy alone was performed in 27 patients.

**Table 1:** Characteristics of the included studies in the review

Authors	Year	Country	Research design	Study groups		Sample size	
				Group 1	Group 2	Group 1	Group 2
Khatib et al 6	2010	France	Retrospective review	TT + BT	TT + UT	45	93
Huang et al 7	2014	China	Retrospective review	TT + UT	TT + BT	73	82
Li et al 8	2019	China	Randomized controlled trial	TT	TT + BT	27	27

Abbreviations: BT, bilateral thymectomy; TT, total thyroidectomy; UT, unilateral thymectomy.

### Demographic Details

The average age of presentation of the patients in either of the subgroups of all the three studies was the fourth decade of life. The mean and median ages have been enumerated in Table 2 . All the studies reported female preponderance among the study subsets with a cumulative female to male ratio of 3.3.

**Table 2:** Patient demographics and clinical characteristics reported in the included studies

Variable	Khatib et al 6		Huang et al 7		Li et al 8	
	TT + BT *(n* = 45)	TT + UT ( *n* = 93)	TT + UT ( *n* = 73)	TT + BT ( *n* = 82)	TT ( *n* = 27)	TT + BT ( *n* = 27)
**(I) Demographic**						
Age (years)	46 (17–85) a	45 (6–78) a	48.1 ± 10.7 b	48.7 ± 10.4 b	45.3 ± 7.8 b	47.3 ± 11.6 b
Gender (M/F)	15/38	27/66	11/62	17/65	5/22	6/21
BMI (kg/m ^2^ )	NA	NA	NA	NA	24.6 ± 4.06	25.2 ± 3.19
**(II) Tumor factors**						
Size (mm)	11.2 (< 1–55) c	18.1 (< 1–55) c	27.6 ± 12.3 b	25 ± 12.0 b	9.78 ± 6.4 b	8.85 ± 4.9 b
Histology						
Papillary	42	75	73	82	27	27
Follicular	0	2	Nil	Nil	Nil	Nil
Medullary	3	17	Nil	Nil	Nil	Nil
Risk stratification (MACIS) (< 6/> 6)	NA	NA	7/73	16/82	NA	NA

Abbreviations: BT, bilateral thymectomy; TT, total thyroidectomy; SD, standard deviation; UT, unilateral thymectomy.

a Median (range)

b Mean ± SD

c Average (range)

### Tumor Characteristics

The average tumor size of various subgroups in the three studies analyzed has been enumerated in Table 2 . Variability in mean tumor size was noted among various studies; however, no statistically significant difference was noted in the individual subgroups.

### Operation Related Factors

Table 3 displays the operative parameters and surgical outcomes reported in the included studies. Only one study by Li et al 8 documented their mean intraoperative time, and there was no significant difference noted when TT alone was performed versus when combined with bilateral thymectomy. Huang et al 7 reported parathyroid autotransplantation rates of 5.1 ± 1.5 and 5.2 ± 1.3 among the patients undergoing unilateral and bilateral thymectomy, respectively, along with TT. However, there was no statistically significant difference between the two subgroups ( *p* = 0.657). Similar results with no statistically significant difference were reported by Khatib et al 6 in their study of 138 patients. Li et al 8 reported that the rates of incidental parathyroidectomy were more common in patients undergoing thymectomy than in those who did not (29.6% vs. 7.4%, *p* = 0.038).

**Table 3:** Operative parameters and surgical outcomes reported in the included studies

Variable	Khatib et al 6		Huang et al 7		Li et al 8	
	TT + BT ( *n* = 45)	TT + UT ( *n* = 93)	TT + UT ( *n* = 73)	TT + BT ( *n* = 82)	TT ( *n* = 27)	TT + BT ( *n* = 27)
Operative duration (min) ± SD	NA	NA	NA	NA	129.52 ± 31.73	121.30 ± 33.10
Hospital stay (days)	NA	NA	NA	NA	6.22 ± 1.97	6.93 ± 2.17
Parathyroid removal/ transplant rates	7 (15.6%)	8 (8.6%)	5.1 ± 1.5	5.2 ± 1.3	2 (7.4%)	8 (29.6%) ( *p* = 0.038)
POD1 PTH levels (pg/ml)	NA	NA	NA	NA	25.46 ± 14.72	11.07 ± 6.03 ( *p* < 0.001)
Vocal fold palsy						
Permanent	NA	NA	NA	NA	0 (0%)	1 (3.7%)
Transient	NA	NA	NA	NA	5 (18.5%)	3 (11.1%)
Hypoparathyroidism						
Permanent	1 (2.2%)	0 (0%)	0 (0%)	3 (3.6%)	0 (0%)	4 (14.8%)
Transient	16 (35.5%)	10 (10.7%)	10 (13.7%)	43 (52.4%)	7 (25.9%)	19 (70.4%)

Abbreviations: TT, total thyroidectomy; UT, unilateral thymectomy; BT, bilateral thymectomy

### Surgical Outcomes and Oncological Completeness

**Fig. 3:**
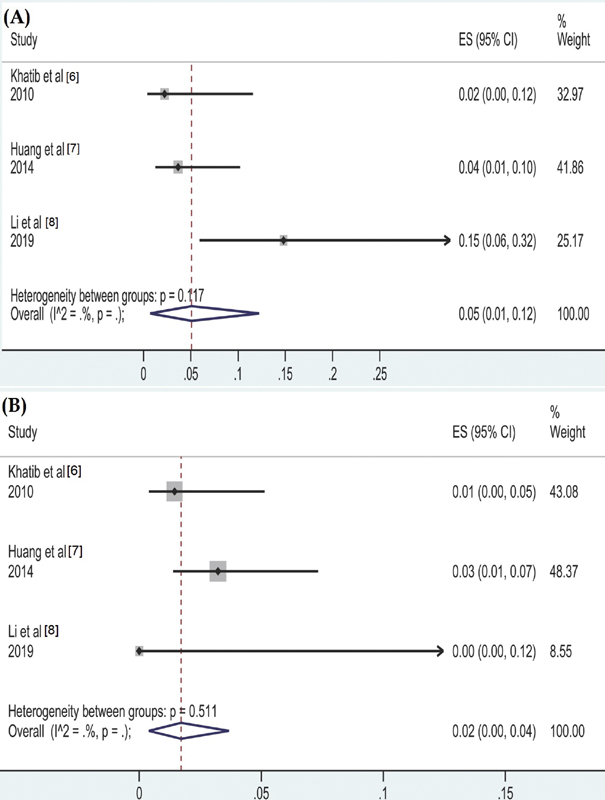
( **A** ) Pooled analysis of postoperative permanent hypocalcemia in patients undergoing bilateral thymectomy during central compartment lymph node dissection (CLND) ( **B** ) Pooled analysis of thymic metastasis in patients undergoing thymectomy during CLND

The pooled frequency of transient hypocalcemia in unilateral and bilateral thymectomy was 12% (95%CI, 7%–17%) and 51% (95% CI 43%–59%), respectively. The pooled frequency of permanent hypocalcemia in bilateral thymectomy was 5% (95% CI 1%–12%) ( Fig. 3A ). Li et al 8 reported that the rates of transient vocal fold palsy between the thymus preservation and bilateral thymectomy groups were comparable (18.5% vs. 11.1%, *p* = 0.704). Permanent vocal cord palsy was reported in one patient in the bilateral thymectomy group due to the violation of the recurrent laryngeal nerve. With regard to ^131^ I treatment, there was no significant difference in preablation serum thyroglobulin levels between the thymus preservation and bilateral thymectomy groups (1.82 ± 2.18 vs. 1.42 ± 1.56, *p* = 0.775).

### Pathological Outcomes

Table 4 displays the pathological outcomes reported in the included studies. The pooled frequency of thymic metastasis was a mere 2% (95%CI, 0%-4%) in patients undergoing either unilateral or bilateral thymectomy ( Fig. 3B ). Huang et al 7 reported a total of five cases of thymic metastases situated in the ipsilateral thymus. No contralateral thymic metastases were found in either group. Likewise, Khatib et al 6 also reported two cases of thymic metastases in the bilateral thymectomy group, both of which were situated in the ipsilateral thymus on pathological examination.

**Table 4:** The pathological outcomes reported in the included studies

Variable	Khatib et al 6		Huang et al 7		Li et al 8	
	TT + BT ( *n* = 45)	TT + UT ( *n* = 93)	TT + UT ( *n* = 73)	TT + BT ( *n* = 82)	TT ( *n* = 27)	TT + BT ( *n* = 27)
**Lymph node metastases**						
Central compartment	20 (44.5%) (all PTC)	53 (57%) (PTC = 44; MC = 9)	NA	NA	14 (51.9%)	14 (51.9%)
Lateral ipsilateral	13 (28.9%) (all PTC)	37(39.8%) (PTC = 31; MC = 6)	NA	NA	NA	NA
Lateral contralateral	1 (2.2%) (all PTC)	8 (8.6%) (PTC = 6; MC = 2)	NA	NA	NA	NA
Thymic metastases	2 (4.4%)	0 (0%)	2 (2.7%)	3 (3.6%)	Nil	Nil

Abbreviations: BT, bilateral thymectomy; MC, medullary carcinoma; PTC, papillary thyroid carcinoma; TT, total thyroidectomy; UT, unilateral thymectomy.

## Discussion

The thymus is a specialized lymphoid organ located in the anterior superior mediastinum associated with T-cell maturation and is critical to the adaptive immune system. The presence of a blood thymic barrier restrains a direct contact of unwanted antigens and tumor cells with the thymus and thereby prevents metastasis. However, on precise analysis, structural variation in the cortex and medulla of the organ explains the variable robustness of this barrier, which provides a possibility for metastasis. 9 The current systematic review shows that the pooled frequency of thymic metastasis was a mere 2% in patients with DTC undergoing either unilateral or bilateral thymectomy.

Moreover, the present review also highlights that routine thymectomy (unilateral or bilateral) does not improve lymph node yield in patients undergoing CLND. However, a cervical extension of the thymus is frequently encountered in about two-thirds of children and young adults as a direct continuation of mediastinal thymic tissue. 10 This cervical thymic extension may warrant selective resection in a particular patient to achieve optimum lymph nodal clearance during CLND. A description of its extent in the radiology report can serve as a useful guide to the surgeon contemplating CLND.

The results of our pooled analysis show that unilateral and bilateral thymectomy was associated with high chances of transient hypocalcemia, albeit the pooled frequency of permanent hypocalcemia was low. Lin et al 11 presented results of their retrospective cohort study including 3186 patients who underwent thyroidectomy and reported that TT and CLND were independent risk factors for incidental parathyroidectomy and resultant postoperative hypocalcemia. The low rates of permanent hypoparathyroidism can be attributed to the fact that postoperative parathyroid gland function mainly depends on the number of parathyroid glands remaining in situ after thyroidectomy. 12 The resultant hypocalcemia serves as a trigger for the remaining parathyroid glands to maintain the serum parathyroid hormone (PTH) values within the normal range. 13 Wide variation in the incidence of hypocalcemia across different studies may be attributed to the surgeons' experience, surgical techniques, and the annual volume of thyroidectomies at a particular center.

Thyroidectomy performed for carcinoma is a high-risk operation, as the posterior capsule is radically dissected with the gland, placing the parathyroid glands as well as the recurrent laryngeal nerve at higher risk of injury. 14 An additional thymectomy in such scenarios definitively increases the risk of transient as well as permanent hypocalcemia in the postoperative period. However, the expertise and experience of the operating surgeon undoubtedly remains a strong predictor of final surgical outcomes. An association between aggressive treatment protocols and deterioration in health-related quality of life (HRQoL) scores has been reported in thyroid cancer survivors by various authors. The recent ATA guidelines have also emphasized the need for developing validated patient-reported outcome measurement tools for assessing the factors that have a bearing on the quality of life as a part of research on thyroid cancer survivorship. Goswami et al 15 conducted an online survey of 1,743 thyroid cancer survivors, of which 98% underwent surgery, using a patient-reported outcomes measurement information system (PROMIS) 29-item profile to evaluate their quality of life. The authors found that patient age < 45 years, postoperative hypocalcemia, and dysphonia were among several other factors that were associated with significantly worse HRQoL scores across various PROMIS domains.

Therapeutic CLND for nodal metastases in DTC is well-accepted for cN1 disease. However, controversy surrounds its role in cN0 neck, although acceptable results can be achieved with low morbidity by an experienced thyroid surgeon. 3 There is a lack of robust data on survival outcomes with limited literature favoring prophylactic dissection in view of improved disease-specific survival (DSS), 16 local recurrence, 17 18 and posttreatment Tg levels. 17 19 In the light of the paucity of literature on any additional survival benefit conferred by the extensive resections in the central compartment, the question is raised as to whether increasing the morbidity of resection by incorporating the thymus in the resection specimen is a risk worth taking?

The main limitation of this systematic review was the limited number of studies addressing the issue of routine thymectomy during the clearance of the central compartment in DTC. Moreover, two of the three studies included in the review were retrospective observational studies generating low-level evidence. One of them included 20 patients with medullary thyroid cancer in their study. 6 The third study despite being a randomized control trial was a single institute study with a limited sample size to evaluate the oncological completeness and a short follow-up period to obtain convincing data on recurrence and metastasis. Lack of data in the selected studies on the association of thymic metastases with the tumor stage, extra thyroid extension, number of involved and sampled lymph nodes, size of the largest involved lymph node, extranodal extension, and vascular invasion precluded establishing any statistically significant correlation. Moreover, no meaningful correlation in the lymph node yield following a CNLD could be calculated across various groups (TT vs. bilateral thymectomy vs. no thymectomy) due to unavailability or scarcity of the relevant data in the selected studies. Thus, large randomized controlled trials with long-term follow-up are needed to generate reliable literature in this context. However, a relatively indolent nature of the disease precludes ideal treatment research protocols to be undertaken.

## Conclusion

This systematic review elucidates that the literature on the role of thymectomy during CLND in patients with DTC is sparse. As the thymectomy during CLND does not confer any additional oncological benefit and is associated with a high risk of postoperative hypocalcemia, thymic preservation must be considered by the operating surgeons, barring the situations involving multiple metastatic nodes close to the thymus and warranting selective thymectomy.

## References

[1] Bray F Ferlay J Soerjomataram I Siegel R L Torre L A Jemal A Global cancer statistics 2018: GLOBOCAN estimates of incidence and mortality worldwide for 36 cancers in 185 countries CA Cancer J Clin 2018 68 06 394 424 30207593 10.3322/caac.21492

[2] Haugen B R Sawka A M Alexander E K Bible K Caturegli P Doherty G The ATA guidelines on management of thyroid nodules and differentiated thyroid cancer task force review and recommendation on the proposed renaming of eFVPTC without invasion to NIFTP Thyroid 2017 27 481 483 28114862 10.1089/thy.2016.0628

[3] Haugen B R Alexander E K Bible K C 2015 American Thyroid Association management guidelines for adult patients with thyroid nodules and differentiated thyroid cancer: the American Thyroid Association guidelines task force on thyroid nodules and differentiated thyroid cancer Thyroid 2016 26 01 1 133 26462967 10.1089/thy.2015.0020 PMC4739132

[4] American Thyroid Association Surgery Working Group American Association of Endocrine Surgeons American Academy of Otolaryngology-Head and Neck Surgery American Head and Neck Society Carty S E Cooper D S Doherty G M Consensus statement on the terminology and classification of central neck dissection for thyroid cancer Thyroid 2009 19 11 1153 1158 19860578 10.1089/thy.2009.0159

[5] Boyd J D Development of the thyroid and parathyroid glands and the thymus Ann R Coll Surg Engl 1950 7 06 455 471 14790564 PMC2238469

[6] El Khatib Z Lamblin J Aubert S Is thymectomy worthwhile in central lymph node dissection for differentiated thyroid cancer? World J Surg 2010 34 06 1181 1186 20094884 10.1007/s00268-009-0363-1

[7] Huang D-P Ye X-H Xiang Y-Q Zhang X-H Thymectomy in central lymph node dissection for papillary thyroid cancer Int J Clin Exp Med 2014 7 04 1135 1139 24955195 PMC4057874

[8] Li W Wang B Jiang Z G Feng Y J Zhang W Qiu M The role of thymus preservation in parathyroid gland function and surgical completeness after bilateral central lymph node dissection for papillary thyroid cancer: A randomized controlled study Int J Surg 2019 65 1 6 30818068 10.1016/j.ijsu.2019.02.013

[9] Kendall M D Functional anatomy of the thymic microenvironment J Anat 1991 177 1 29 1769884 PMC1260410

[10] Costa N S Laor T Donnelly L F Superior cervical extension of the thymus: a normal finding that should not be mistaken for a mass Radiology 2010 256 01 238 242 20505060 10.1148/radiol.10091792

[11] Lin Y S Hsueh C Wu H Y Yu M C Chao T C Incidental parathyroidectomy during thyroidectomy increases the risk of postoperative hypocalcemia Laryngoscope 2017 127 09 2194 2200 28121013 10.1002/lary.26448

[12] Lorente-Poch L Sancho J J Ruiz S Sitges-Serra A Importance of in situ preservation of parathyroid glands during total thyroidectomy Br J Surg 2015 102 04 359 367 25605285 10.1002/bjs.9676

[13] Hermann M Ott J Promberger R Kober F Karik M Freissmuth M Kinetics of serum parathyroid hormone during and after thyroid surgery Br J Surg 2008 95 12 1480 1487 18991283 10.1002/bjs.6410

[14] Del Rio P Rossini M Montana C M Postoperative hypocalcemia: analysis of factors influencing early hypocalcemia development following thyroid surgery BMC Surg 2019 18 01 25 31074401 10.1186/s12893-019-0483-y PMC7402573

[15] Goswami S Peipert B J Mongelli M N Clinical factors associated with worse quality-of-life scores in United States thyroid cancer survivors Surgery 2019 166 01 69 74 30898373 10.1016/j.surg.2019.01.034

[16] Barczyński M Konturek A Stopa M Nowak W Prophylactic central neck dissection for papillary thyroid cancer Br J Surg 2013 100 03 410 418 23188784 10.1002/bjs.8985

[17] Popadich A Levin O Lee J C A multicenter cohort study of total thyroidectomy and routine central lymph node dissection for cN0 papillary thyroid cancer Surgery 2011 150 06 1048 1057 22136820 10.1016/j.surg.2011.09.003

[18] Hartl D M Mamelle E Borget I Leboulleux S Mirghani H Schlumberger M Influence of prophylactic neck dissection on rate of retreatment for papillary thyroid carcinoma World J Surg 2013 37 08 1951 1958 23677562 10.1007/s00268-013-2089-3

[19] Sywak M Cornford L Roach P Stalberg P Sidhu S Delbridge L Routine ipsilateral level VI lymphadenectomy reduces postoperative thyroglobulin levels in papillary thyroid cancer Surgery 2006 140 06 1000 1005, discussion 1005–1007 17188149 10.1016/j.surg.2006.08.001

